# Long-term patient-reported neurocognitive and functional outcomes after tick-borne encephalitis: a prospective controlled multicenter cohort study

**DOI:** 10.1007/s00415-026-14095-3

**Published:** 2026-08-27

**Authors:** Hilde Skudal, Åslaug Rudjord Lorentzen, Tore Stenstad, Else Quist-Paulsen, Børre Fevang, Keson Jaioun, Malin Veje, Hege Kersten, Randi Eikeland, Jens Egeland

**Affiliations:** 1 Department of Infectious Diseases, Telemark Hospital Trust, Skien, Norway; 2 Faculty of Medicine, University of Oslo, Institute of Clinical Medicine, Oslo, Norway; 3 Department of Neurology, Sørlandet Hospital Trust, Kristiansand, Norway; 4 Sørlandet Hospital Trust, National Center on Tick-borne Diseases, Kristiansand, Norway; 5 Department of Infectious Diseases, Vestfold Hospital Trust, Tønsberg, Norway; 6 Department of Microbiology, Oslo University Hospital, Oslo, Norway; 7 Department of Rheumatology Dermatology and Infectious Diseases, Section of Clinical Immunology and Infectious Diseases, Oslo University Hospital, Oslo, Norway; 8 Department of Research, Telemark Hospital Trust, Skien, Norway; 9 Department of Infectious Diseases, Sahlgrenska University Hospital, Region Västra Götaland, Gothenburg, Sweden; 10 Department of Infectious Diseases, University of Gothenburg, Institute of Biomedicine, Gothenburg, Sweden; 11 Faculty of Health and Sport Sciences, University of Agder, Grimstad, Norway; 12 Division of Mental Health and Addiction, Vestfold Hospital Trust, Tønsberg, Norway; 13 Department of Psychology, University of Oslo, Oslo, Norway

**Keywords:** Tick-borne encephalitis, Central nervous system infections, Long-term outcome, Cognitive symptoms, Cohort studies, Patient-reported outcome measures

## Abstract

**Objective:**

To characterize cognitive symptoms the first year after tick-borne encephalitis (TBE).

**Methods:**

We conducted a controlled, multicenter cohort study of 109 hospitalized patients ≥16 years with confirmed TBE in Norway. At 3 and 12 months after admission, patients completed BRIEF-A (Global Executive Composite (GEC) and subscales), Everyday Memory Questionnaire-Revised (EMQ-R), and Rivermead Post-Concussion Symptoms Questionnaire (RPQ), assessing executive functioning, memory, and post-TBE symptoms (higher scores indicate more difficulties). The primary outcomes were within-patient change in questionnaire scores from 3 to 12 months, and 12-month scores in patients compared to controls. The secondary outcomes were BRIEF-A subscale scores and proportion with clinically significant impairment. Covariates included in the analyses were age, sex, comorbidities, acute disease severity, anxiety, and depression.

**Results:**

There were no significant changes from 3 to 12 months. At 12 months, scores were within the normal range, but patients had higher EMQ-R and RPQ than controls (*p*<0.001). BRIEF-A GECs did not differ, but patients had higher working memory subscale scores (*p*=0.002). These differences persisted after adjustment for anxiety and depression. Clinically significant impairment was more frequent in patients (odds ratios 2.29–8.19). Comorbidities, acute disease burden, anxiety, depression and female sex were associated with unfavorable 12 month outcome.

**Conclusion:**

TBE patients have an overall good one-year prognosis, but a minority—characterized by higher acute disease burden and comorbidities—have persistent cognitive difficulties that may warrant follow-up. Anxiety and depression were related to unfavorable prognosis but did not account for the cognitive differences between patients and controls.

**Trial registration.:**

Project #2,296,959**—**The NOrwegian Tick-borne Encephalitis Study—NOTES**.** An Observational Study on Clinical Features, Long-term Outcomes and Immune Characteristics—Cristin.

**Supplementary Information:**

The online version contains supplementary material available at https://doi.org/10.1007/s00415-026-14095-3.

## Introduction

Tick-borne encephalitis (TBE) is a neurotropic viral disease caused by the tick-borne encephalitis virus (TBEV), primarily affecting the central nervous system (CNS) and represents one of the most important arthropod-borne neurological infections in Europe [1]. Clinical manifestations vary, ranging from flu-like symptoms to central nervous system involvement. Neurological manifestations include meningitis, encephalitis, myelitis, radiculitis, or combinations of these. Mainly three different subtypes of the virus cause human disease [2], and in Norway only strains of the European subtype (TBEV-Eu) of the virus have been detected [3].

Over the last decade, the incidence of TBE has increased across endemic countries in Europe, with geographic expansion into previously non-endemic regions [4, 5].

Similar trends have been observed in Scandinavia, including Norway [6].

Although the acute mortality associated with TBE caused by TBEV-Eu is relatively low (0.5–2%) [7, 8], recent studies demonstrate a substantial burden of long-term morbidity [9–11]. Across the limited number of available European cohorts, neurological and cognitive sequelae are reported in about 40% of patients [12, 13], including after apparently mild TBE [11], underscoring that TBE is a condition with clinically significant consequences well beyond the acute phase.

However, the long-term consequences of TBE remain incompletely defined. The relationship between subjective symptoms and objective impairment is unclear, and predictors of persistent sequelae are insufficiently understood. In addition, mild psychiatric symptoms such as anxiety and depression frequently follow infectious disease and may confound or exacerbatecognitive complaints, but their contribution in TBE populations has not been systematically evaluated.

Furthermore, there is no consensus on how TBE sequelae should be defined or reported [14]. Several critical gaps remain. First, the course of subjective cognitive symptoms the first year following infection is scarcely investigated prospectively [11, 15, 16]. Second, few studies have characterized differences between TBE patients and control participants [9, 11, 17]. Third, the proportion of individuals with clinically significant impairment has not been established. Finally, the factors underlying prolonged recovery after TBE remain insufficiently understood. Potential contributors include premorbid health status, acute disease severity, and co-occurring psychiatric symptoms such as anxiety and depression, as well as differential influence of sex and age. In addition, the present study includes exploratory analyses of structural brain abnormalities, work participation, and persisting objective neurological deficits, as supplementary indicators of long-term outcome after TBE.

Previous studies have aimed to identify predictors of poor outcome in TBE. Increasing age has been reported to be a risk factor for adverse outcomes in some studies [10, 18], whereas the roles of premorbid health and disease severity, although biologically plausible markers of vulnerability, have yielded inconsistent results [10, 11, 17]. Male sex has been associated with the risk of severe acute TBE at admission [19], but does not consistently predict long-term sequelae [10]. These prior, and partly inconsistent, findings, together with the biological plausibility of these factors, provide a rationale for examining sex, age, premorbid health status, disease severity, and co-occurring mild psychiatric symptoms as potential mediators of long-term outcome after TBE.

To address these gaps, the objectives of this study were to:

(i) assess the trajectory of patient-reported cognitive and functional symptoms from 3 to 12 months after TBE;
(ii) compare 12-month self-reported cognitive outcomes between TBE patients and comparable controls;
(iii) evaluate the clinical relevance of any observed differences; and
(iv) identify predictors of persistent symptoms, including premorbid health status, age, sex, disease severity, and to examine whether symptoms of anxiety and depression mediate subjective cognitive complaints.

## Methods

### Study design and setting

We conducted a multicenter prospective cohort study (Norwegian Tick-borne Encephalitis Study [NOTES]) at the three hospitals serving the TBE-endemic regions of Norway. Patients with confirmed TBE were assessed during hospitalization and at 3 and 12 months after admission. At both follow-up visits, patients completed patient-reported outcome measures (PROMs) as described below. This article adheres to the STROBE guidelines for reporting observational cohort studies [20].

### Study population

#### Patients

Patients for the present study were recruited from the NOTES cohort [21]. Hospitalized TBE patients admitted between January 2018, and December 2022 were eligible for inclusion. The sample size in this follow-up study was determined by the number of eligible patients enrolled in the NOTES cohort.

Inclusion criteria were:

(i) confirmed tick-borne encephalitis (TBE) according to the ECDC case definition [22], defined as clinical signs of central nervous system infection (e.g., meningitis, meningoencephalitis, meningoencephalomyelitis, encephaloradiculitis, or meningomyelitis) plus at least one laboratory criterion: TBEV-specific IgM and IgG in serum, TBEV-specific IgM in cerebrospinal fluid (CSF), IgG seroconversion in paired sera, or detection of TBEV RNA by real-time PCR;
(ii) age ≥16 years
(iii) verified or suspected tick exposure in southeastern Norway; and
(iv) hospitalization at a participating hospital

Vaccinated patients were included if TBEV infection was confirmed by intrathecal TBEV-specific IgG or IgM production, detection of TBEV-specific IgM in CSF, or—when both IgG and IgM were detected in serum—if ≥10 months had elapsed since the last vaccination dose [23], together with compatible clinical findings and CSF pleocytosis (> 5 × 10⁶ cells/L).

#### Control group

We recruited demographically comparable controls from patients’ social networks, including family members, friends, and colleagues, who were identified by the patients and invited by the study team. This approach intended to yield a close demographic similarity between cases and controls [24]. Controls independently completed the same questionnaires as the patients and additionally answered a questionnaire on demographic information and medical history. Clinical investigations were not performed in the controls.

#### Data collection

Specialists in neurology and infectious diseases at the participating centers identified and investigated eligible patients at baseline and the follow-up visits. Demographic data were collected during the hospital stay for acute TBE. Information on work participation and sick leave was obtained by patient self-report at the follow-up visits. Magnetic resonance imaging (MRI) and computed tomography (CT) of the brain were performed during the acute phase according to routine clinical indications. For the present study, MRI and CT reports were reviewed, and pathological findings were coded dichotomously (presence vs. absence of findings suggestive of encephalitis) and included as a potential predictor in the regression analyses of long-term-patient-reported outcomes.

#### Composite clinical score (CCS)

Patient symptoms and objective findings related to TBE were assessed and registered at baseline, 3 to 12 months using a Composite Clinical Score (CCS) consisting of 17 items of symptoms and 22 objective neurological findings. The CCS is described in detail in a previous publication [21]. Each item was scored from 0 to 2 points, with 0 indicating no symptoms, 1 indicating mild symptoms without influence on daily life, and 2 indicating severe symptoms with influence on daily life, giving a maximum score of 78.

#### Disease classification

During the acute phase, patients were categorized into three severity groups, using a classification system previously used in TBE studies [11, 17]. Mild disease was defined by meningeal symptoms including fever, headache, nausea, vomiting, neck stiffness, and photo- and/or phonophobia, with normal or non-performed electroencephalography (EEG). Moderate disease was defined by mildly altered consciousness and/or focal neurological signs, whereas severe disease was defined by multifocal neurological symptoms and/or pronounced signs of encephalitis with more substantial alteration of consciousness. Cognitive symptoms were assessed clinically through observation and categorized as mental slowness, confusion, or altered level of consciousness. Clinical features compatible with myelitis were evaluated based on neurological examination.

#### Patient-reported outcome measures (PROMs)

Validated, standardized, specific questionnaires for evaluating sequelae after TBE do not exist. Therefore, we used established and widely applied PROMs to evaluate cognitive executive functions, memory problems, and symptom burden after TBE. Participants completed the questionnaire in either a paper-based or digital format. The following PROMs were included:

#### The behavior rating inventory of executive function

The Behavior Rating Inventory of Executive Function-Adult version (BRIEF-A) [25] is a 75-item questionnaire assessing everyday executive functioning. It is one of the most widely used self-reported instruments for evaluating executive function in daily life [26] and is considered sensitive to intra-individual changes [27]. The questionnaire consists of nine subscales (Inhibit, Shift, Emotional Control, Self-Monitor, Initiate, Working Memory, Plan/Organize, Task Monitor, Organization of Materials). Together, these subscales form the Global Executive Composite (GEC), an overall index of executive functioning in BRIEF-A. We used the BRIEF-A GEC as the primary outcome measure and the nine subscales as the secondary outcomes (Table 2). Both the GEC and the subscale scores are standardized T-scores with a normative mean of 50 (SD = 10). T-scores are capped at a maximum of 100, with scores ≥65 indicating clinically significant executive difficulties.

#### The everyday memory questionnaire

Everyday memory was assessed using the Everyday Memory Questionnaire-Revised (EMQ-R) [28], a validated measure of memory difficulties. The EMQ-R consists of 13 self-reported items assessing the frequency of everyday memory lapses, each rated on a Likert scale ranging from 0 to 4 (from once or less in the last month to once or more often per day), giving a total score of 0–52, where higher scores indicate greater memory difficulties. We used the total EMQ-R total score as an outcome (Table 2). In this article, EMQ-R is used to denote the EMQ-R total score. Clinically relevant memory impairment was defined as EMQ-R ≥19, corresponding to approximately 1.5 SD above the mean total score for the control group (Table 2).

#### The rivermead post-concussion symptoms questionnaire

The Rivermead Post-Concussion Symptoms Questionnaire (RPQ) [29] is a widely used measure of symptom burden after mild brain injury. It captures a broad range of physical, cognitive, and psychological symptoms relevant for TBE patients and not fully covered by executive-function measures in the BRIEF-A. RPQ has previously been used to evaluate long-term outcomes after TBE in children [30]. It consists of 16 symptoms graded for severity over the past 24 h on a 0–4 Likert scale, yielding a total score of 0–64. Higher scores indicate greater symptom burden. The RPQ is validated [31]. A total score ≥16 was considered indicative of severe symptoms [32]. In this article, RPQ is used to denote the RPQ total score.

#### Covariates

The symptoms of anxiety and depression were assessed with the validated Norwegian version of the Hospital Anxiety and Depression Scale (HADS) [33], which comprises 14 items divided into anxiety (HADS-A) and depression (HADS-D) subscales (score range, 0–21 for each). HADS-A and HADS-D scores were entered as continuous covariates in the multivariate analysis of covariance (MANCOVA) to assess the mediating effect of psychiatric comorbidity on the cognitive outcome measures (i.e. BRIEF-A GEC, EMQ-R, and RPQ). Sex, age, and comorbidity were also included as potential mediators or moderators.

#### Predefined outcomes

The predefined primary and secondary outcomes are summarized in Table 1. The primary outcomes were defined as: (i) change in PROM scores (BRIEF-A GEC score, EMQ-R, RPQ), from 3 to 12 months within the patient group and (ii) PROM scores at 12 months comparing TBE patients and controls. Secondary outcomes were defined as: (i) BRIEF-A subscale T-scores comparing TBE patients and controls, and (ii) the proportion of individuals with clinically relevant impairment, based on predefined cutoff scores.

**Table 1:** Primary and secondary outcome measures

Outcome level	Instrument		Outcome measure(s)		Cutoff for impairments	Role in analysis	
Primary outcome	BRIEF-A	GEC T-score (range 0-100) (mean 50, SD± 10)		T-Scores ≥ 65			Executive functioning in daily life; GEC at 12 months in patients vs. controls, and change from 3 to 12 months
Primary outcome	EMQ-R	EMQ-R Total Score (range 0–52)		Total score ≥ 19			Everyday memory lapses; EMQ-R at 12 months in patients vs controls, and change from 3 to 12 months
Primary outcome	RPQ	RPQ Total Score (range 0–64)		Total score ≥16			Physical, emotional, and cognitive symptom burden; RPQ at 12 months in patients vs. controls, and change from 3 to 12 months
Secondary outcome	BRIEF-A	Subscale scores^a^ T-score (range 0-100) (mean 50, SD ±10);		Clinically relevant impairment; T-score ≥ 65			Detailed profile of executive functioning; BRIEF-A subscale score at 12 months in patients vs. controls, and change from 3 to 12 months
Secondary outcome	BRIEF-A EMQ-R RPQ	Proportion with clinically relevant impairment, based on cut-off scores		Dichotomized using cut-offs above: BRIEF-A T-Scores ≥ 65^b^ EMQ-R Total score ≥ 19 RPQ Total score ≥ 16			Proportion of patients and controls with clinically relevant difficulties in executive functioning, everyday memory, and symptom burden

*BRIEF-A* behavior rating inventory of executive function–adult version, *GEC* global executive composite, *EMQ-R* everyday memory questionnaire–revised, *RPQ* rivermead post-concussion symptoms questionnaire

^a^BRIEF-A Subscale scores: Inhibit, Shift, Emotional Control, Self-Monitor, Initiate, Working Memory, Plan/Organize, Task Monitor, Organization of Materials

^b^clinically relevant impairment (T-scores ≥ 65) was analyzed only for the BRIEF-A Working Memory subscale as this was the only subscale showing a statistically significant difference between patients and controls

Higher scores reflect greater symptom burden

### Statistical analysis

Continuous variables were assessed for normality and multivariate normality using the Shapiro–Wilk test. For normally distributed variables, means and standard deviations (SD) are reported; for non-normally distributed variables, medians, interquartile ranges (IQR), and ranges are presented. Categorical variables are summarized as frequencies and percentages. Differences in proportions for independent categorical variables were evaluated using Pearson’s chi-square test or Fisher’s exact test, as appropriate.

*Student’s t*-test or the Mann–Whitney U test, as appropriate, was used to compare independent continuous variables between controls and TBE patients at 12 months’ follow-up. Differences in paired dependent continuous variables between 3- and 12-month-follow-ups were evaluated using the paired t-test or Wilcoxon signed-rank test, as appropriate; these analyses were restricted to patients with complete data at both time points. Effect sizes (Cohen’s d) were calculated for changes from 3 to 12 months and for differences between patients at 12 months and controls.

A one-way multivariate analysis of variance (MANOVA) was conducted to compare RPQ, EMQ-R, and BRIEF-A GEC T-score (dependent variables) across case and control groups. Assumptions underlying the one-way MANOVA were assessed. To reduce skewness and improve distributional properties, the variables BRIEF-A GEC, RPQ and EMQ-R were square-root transformed. Box’s M test was used to check the assumption of homogeneity of covariance matrices. Variance Inflation Factor (VIF) and Spearman correlations were used to assess multicollinearity among dependent variables; VIF values < 10 and Spearman correlation below 0.90 were taken to indicate no concerning multicollinearity. When a significant multivariate effect was observed in the MANOVA model (unadjusted), follow-up univariate analyses of variance (ANOVA) were conducted to examine group differences for individual dependent variables. Marginal means and standard errors were reported. A Multivariate Analysis of Covariance (MANCOVA) was performed to compare the same dependent variables between case and control groups, adjusting for age, sex, comorbidity, HADS-A and HADS-D scores. MANCOVA assumptions were assessed using procedures analogous to those applied in the MANOVA. In the presence of a significant multivariate effect in the MANCOVA, follow-up univariate analyses of covariance (ANCOVA) were performed for each dependent variable, adjusting for the covariates. Results are presented as adjusted marginal means and standard errors.

Repeated-measures MANOVA (unadjusted) and MANCOVA (adjusted) were performed, using procedures analogous to those described above, to compare change from 3 to 12 months in BRIEF-A GEC T-score, EMQ-R, and RPQ, with and without adjustment for age, sex, comorbidity, disease severity, HADS-A and HADS-D scores. Effect sizes for multivariate tests (one-way MANOVA, one-way MANCOVA, the repeated-measures MANOVA and repeated-measures MANCOVA) are reported as partial *η*^*2*^.

In additional multivariate models, BRIEF-A Working Memory T-score, EMQ-R, and RPQ were entered as dependent variables. Univariate and multivariate linear regression analyses were performed to examine the association between independent variables and outcome variables BRIEF-A GEC T-score, BRIEF-A Working Memory T-score, EMQ-R, and RPQ. Beta coefficients (*β*) with corresponding 95% confidence intervals were reported as measures of effect size. Model assumptions were assessed prior to interpretation and reporting of the results.

Item-level missingness in the questionnaires was assessed; details of missing data and handling procedures for each PROM are provided in the Supplementary Material.

Internal consistency of all PROMs was assessed using Cronbach’s alpha. The corresponding Cronbach’s alpha coefficients for each PROM are reported in the Supplementary Material.

Sensitivity analyses were conducted by repeating the analyses using complete-case data (excluding imputed observations) to assess the impact of imputation on the study findings. Because hypertension and allergy are not included in the Charlson Comorbidity Index and are commonly not treated as comorbidities in comparable studies, we performed sensitivity analyses excluding participants with these conditions to assess the robustness of our findings.

Statistical significance was defined as a level of *p* < 0.05.

All statistical analyses were performed using STATA Statistical Software, Release 18 (StataCorp LLC, College Station, TX, 2023) and RStudio version 2025.09.2 +418 (RStudio, PBC, Boston, MA).

### Potential sources of bias

Several potential sources of bias were considered. Selection bias may have arisen because controls were recruited from patients’ social networks, which could limit representativeness. Sampling bias was possible because some patients were assessed only at 3 or 12 months, leading to partially overlapping 3- and 12-month samples. To minimize sampling bias in the trajectory analyses, only patients who completed both the 3- and 12-month assessments were included, resulting in identical samples at both time points.

### Data availability

Data supporting the findings of this study are available from the corresponding author upon reasonable request from qualified researchers and upon approval by the respective ethics committee.

### Approval, registrations, and patient consents

This study was approved by the Ethics Committee of South-East Norway (Study no. 96505) and by the data protection officers at each participating hospital. All methods were carried out in accordance with the guidelines and regulations of the Declaration of Helsinki [34]. Written informed consent was obtained from all participants.

## Results

**Fig. 1:**
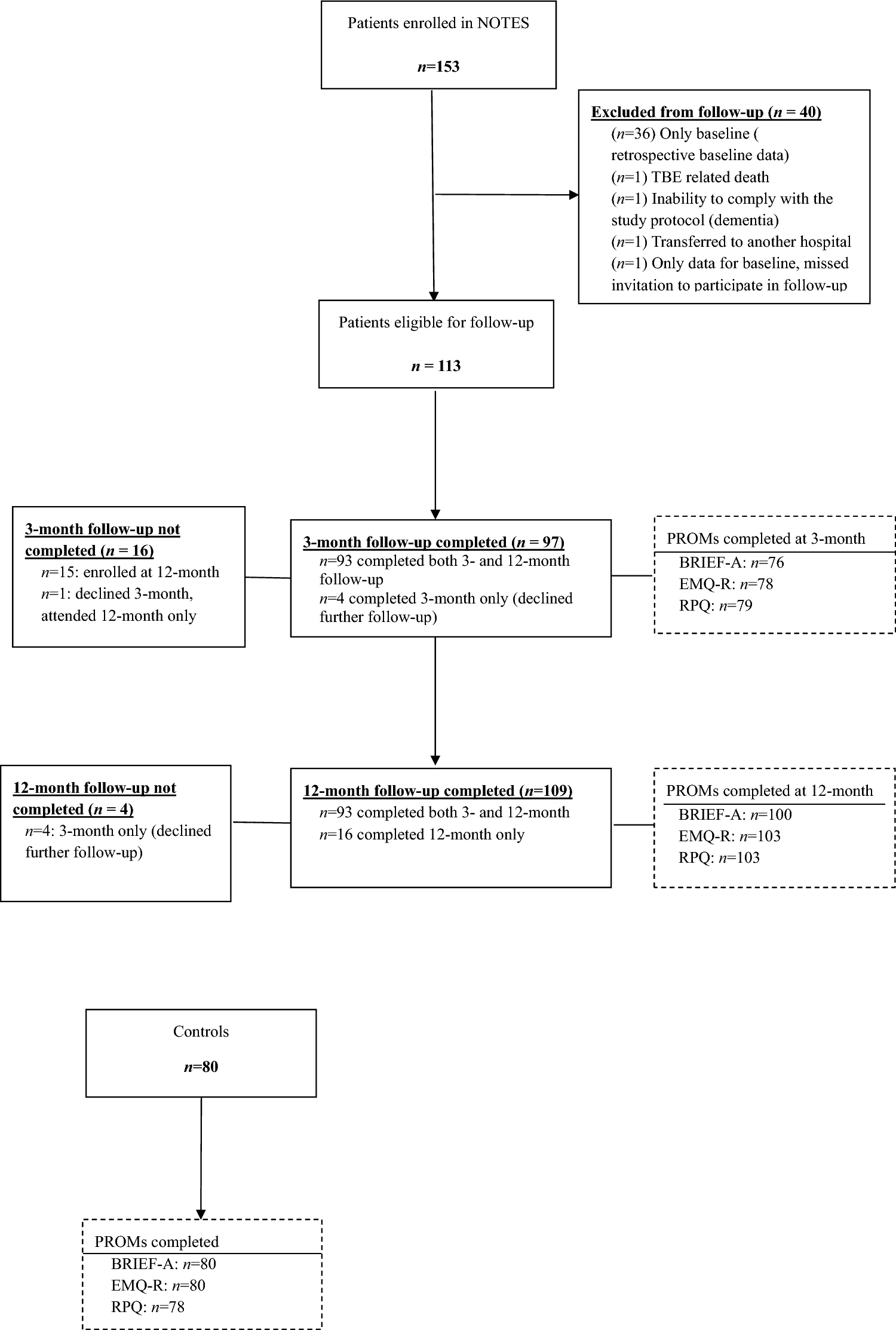
Flow chart of the study population. PROM Patient-reported outcome measure; BRIEF-A Behavior Rating Inventory of Executive Function–Adult Version; EMQ-R Everyday Memory Questionnaire–Revised; RPQ Rivermead Post-Concussion Symptoms Questionnaire

Figure 1 shows the flow of participants through the study, including attendance and response rates for the PROMs. Of 153 patients with verified TBE enrolled in NOTES, 109 completed the 12-month-follow-up and were included in the primary analyses. At 3 months, 97 patients were assessed; 93 completed both 3- and 12-month assessments, 4 only the 3-month visit, and 16 only the 12-month visit. The 16 patients assessed only at 12 months were enrolled too late to be invited to the 3-month visit. For the majority of these patients, baseline data were complete; in a few cases baseline information was obtained retrospectively from medical records. A total of 80 controls completed the PROMs.

Baseline demographic and clinical characteristics of patients and controls are presented in Table 2. The mean age in both groups was in the mid-50 s, with a similar age distribution. A higher proportion of women were included among controls compared to patients (71.3% vs. 61.5%, *p* < 0.001). Educational level, living status and occupational status were largely similar between patients and controls. Any comorbidity was present in about half of patients and in nearly two-thirds of controls, although this difference was not statistically significant. Medication did not differ between groups (Supplementary Table 1). Taken together, these data indicate that, apart from a higher proportion of women among controls, the two groups were broadly comparable in terms of socio-demographic factors and baseline health status.

**Table 2:** Demographic and clinical characteristics of TBE patients and controls

Variable	Patients (*n* = 109)	Controls (*n* = 80)	p-value
Age, years, mean (SD), range	55.1 (15.7), 17–87	53.1 (13.9), 18–81	0.364
Age groups, *n* (%)			0.891ᵃ
16–30	7 (6.4)	5 (6.3)	
31–45	22 (20.2)	20 (25.0)	
46–60	41 (37.6)	28 (35.0)	
>60	39 (35.8)	27 (33.8)	
Sex, *n* (%)			**<0.001**
Female	42 (38.5)	57 (71.3)	
Male	67 (61.5)	23 (28.8)	
Education level, *n* (%)			0.468
≤ 12/13 years	55 (50.5)	40 (50.0)	
> 12/13 years	52 (47.7)	40 (50.0)	
Missing	2 (1.8)	0	
Clinical characteristics			
Any comorbidity, *n* (%)	56 (51.4)	52 (65.0)	0.061
Any medicationᵇ, *n* (%)	57 (52.3)	36 (45.0)	0.322
Occupational status, *n* (%)			0.389
Full/part-time employment	62 (56.9)	56 (70.0)	
Student	3 (2.8)	4 (5.0)	
Age pensioner	25 (22.9)	14 (17.5)	
Disability pension (partial/full)	8 (7.3)	4 (5.0)	
Not in paid employment	3 (1.8)	1 (1.3)	
Sick leave (partial/full)	8 (7.3)	0	
Missing	1 (0.9)	1 (1.3)	
Disease severity (patients only), *n* (%)	—	—	—
Mild	26 (23.9)	—	—
Moderate	70 (64.2)	—	—
Severe	13 (11.9)	—	—

*SD* standard deviation

^a^p-value reported for overall comparison across age categories (χ^2^-test)

^b^Supplementary Table 1 shows details regarding specific comorbidities and medications

Statistically significant results are shown in bold

**Fig. 2:**
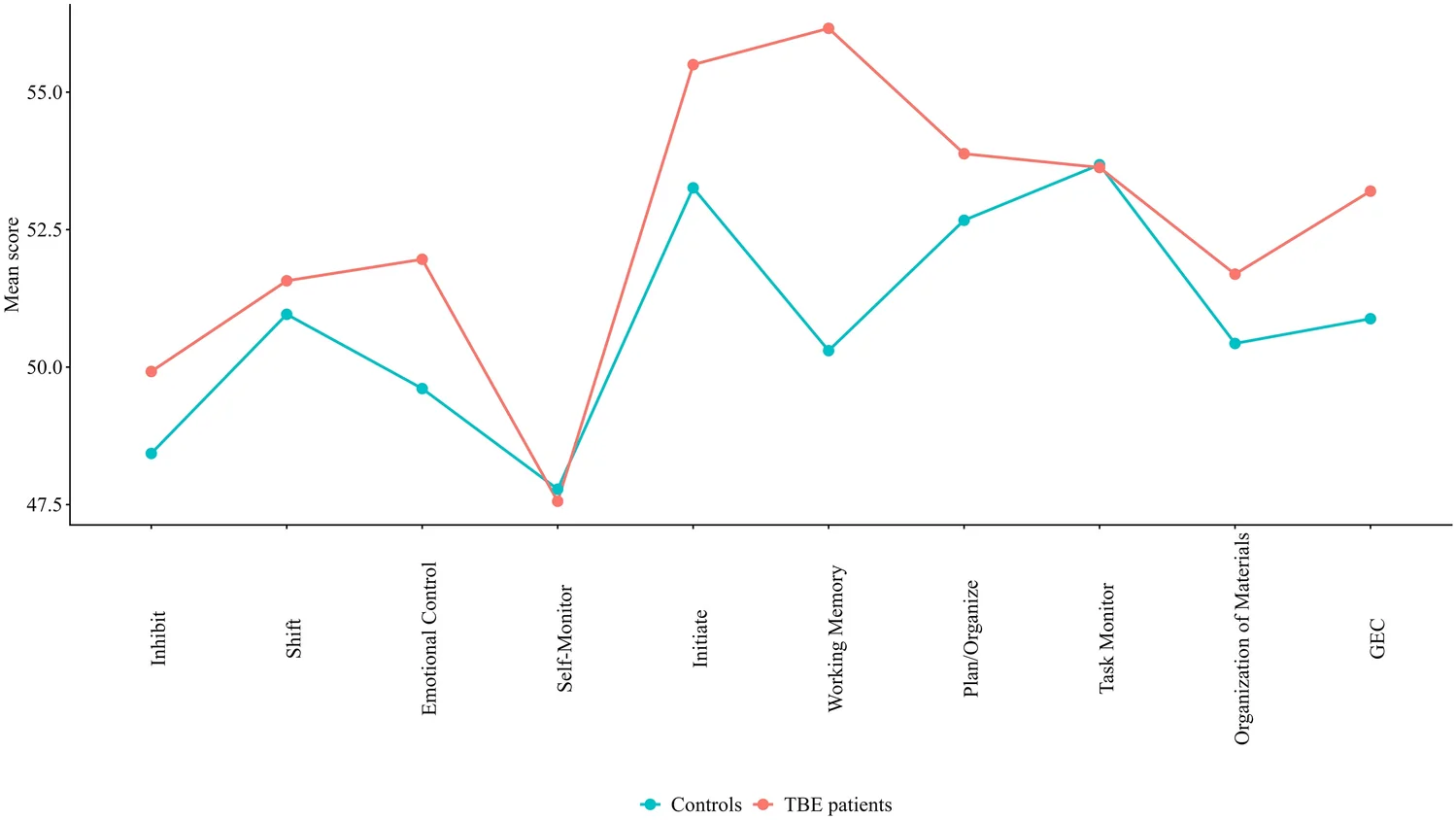
Mean BRIEF-A subscale scores at 12 months in TBE patients (*n* = 100) and controls (*n* = 80). *BRIEF-A* behavior rating inventory of executive function—adult version, Higher scores indicate greater executive difficulties in everyday life

Below, we first describe changes in self-reported cognitive symptoms from 3 to 12 months after TBE, then compare 12-month outcomes between patients and controls, examine the clinical relevance of these differences, and finally explore factors associated with persistent cognitive symptoms at 12 months (Fig. 2).

### Longitudinal analyses of self-reported cognitive outcomes from 3 to 12 months

A total of 71, 73, and 74 patients completed both the 3- and 12-month follow-up assessments for BRIEF-A, EMQ-R, and RPQ, respectively. Within-patient changes in self-reported cognitive and functional symptoms were all non-significant on the BRIEF-A GEC, EMQ-R and RPQ (Supplementary Table 2). Below, we present the results for the primary outcomes (Supplementary Table 2);

### Executive functioning

Mean BRIEF-A GEC T-scores were 53.8 (SD 12.4) at 3 months and 53.2 (SD 12.9) at 12 months (*p* = 0.540). There were no significant within-patient changes for any of the nine BRIEF-A subscales (all *p* ≥ 0.090).

### Everyday memory

Mean EMQ-R were 14.2 (SD 12.0) at 3 months and 13.8 (SD 12.0) at 12 months (*p* = 0.761).

### Symptom burden

Mean RPQ were 17.2 (SD 14.1) at 3 months and 16.8 (15.6) at 12 months (*p* = 0.111).

A repeated-measures MANOVA revealed no significant multivariate effect from 3- to 12-month follow-up on the combined dependent variables (BRIEF-A GEC T-scores, EMQ-R scores, and RPQ scores, *p* = 0.165, partial *η*^2^ = 0.072) (Supplementary Table 3a). This effect remained non-significant in the corresponding repeated-measures MANCOVA after adjustment for the covariates age, sex, comorbidity, disease severity, HADS-A and HADS-D (*p*=0.112). In the fully adjusted multivariate model, age and sex were significant covariates for BRIEF-A GEC, EMQ-R, and RPQ scores, while comorbidity, disease severity, HADS-A and HADS-D were not (Supplementary Table 3a).

### Cognitive symptoms in patients at 12 months versus controls: main effects

The overall multivariate analysis (one-way MANOVA) taking into account all three primary outcome measures, showed a significant group effect, i.e. the patients performing significantly worse than controls (*p* < 0.001, partial *η*^2^ = 0.173). Follow-up univariate ANOVAs (unadjusted) showed that the overall effect was due to significant group differences on EMQ-R and RPQ, but not on BRIEF-A GEC (EMQ-R; *p* =0.001, partial *η*^*2*^ = 0.057, RPQ; *p* < 0.001, partial *η*^*2*^ = 0.142). The patients had higher EMQ-R and RPQ than controls (Marginal mean ±SE; EMQ-R: 3.13 ± 0.16 vs. 2.46 ± 0.18, *p* < 0.001, RPQ: 3.42 ± 0.19, vs. 1.83 ±0.22, *p* < 0.001) (Supplementary Table 3c).

**Fig. 3:**
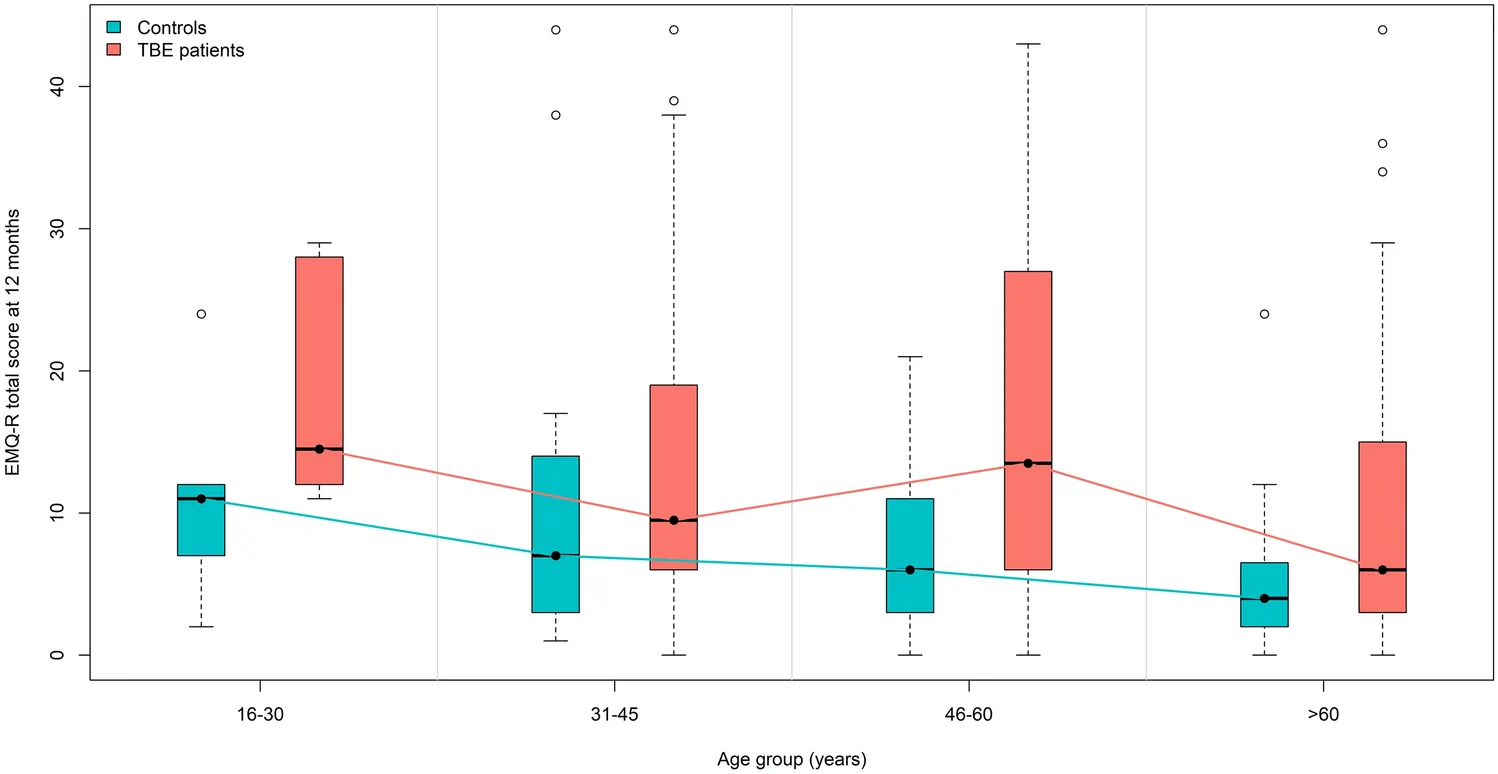
Distribution of EMQ-R scores across age groups in TBE patients (*n* = 103) and controls (*n* = 80), shown as box and whisker plots. The box indicates the interquartile range, the center line the median, whiskers extend to 1.5 × IQR, and points beyond the whiskers represent outliers. *EMQ-R* everyday memory questionnaire – revised, Higher scores indicate greater executive difficulties in everyday life

**Fig. 4:**
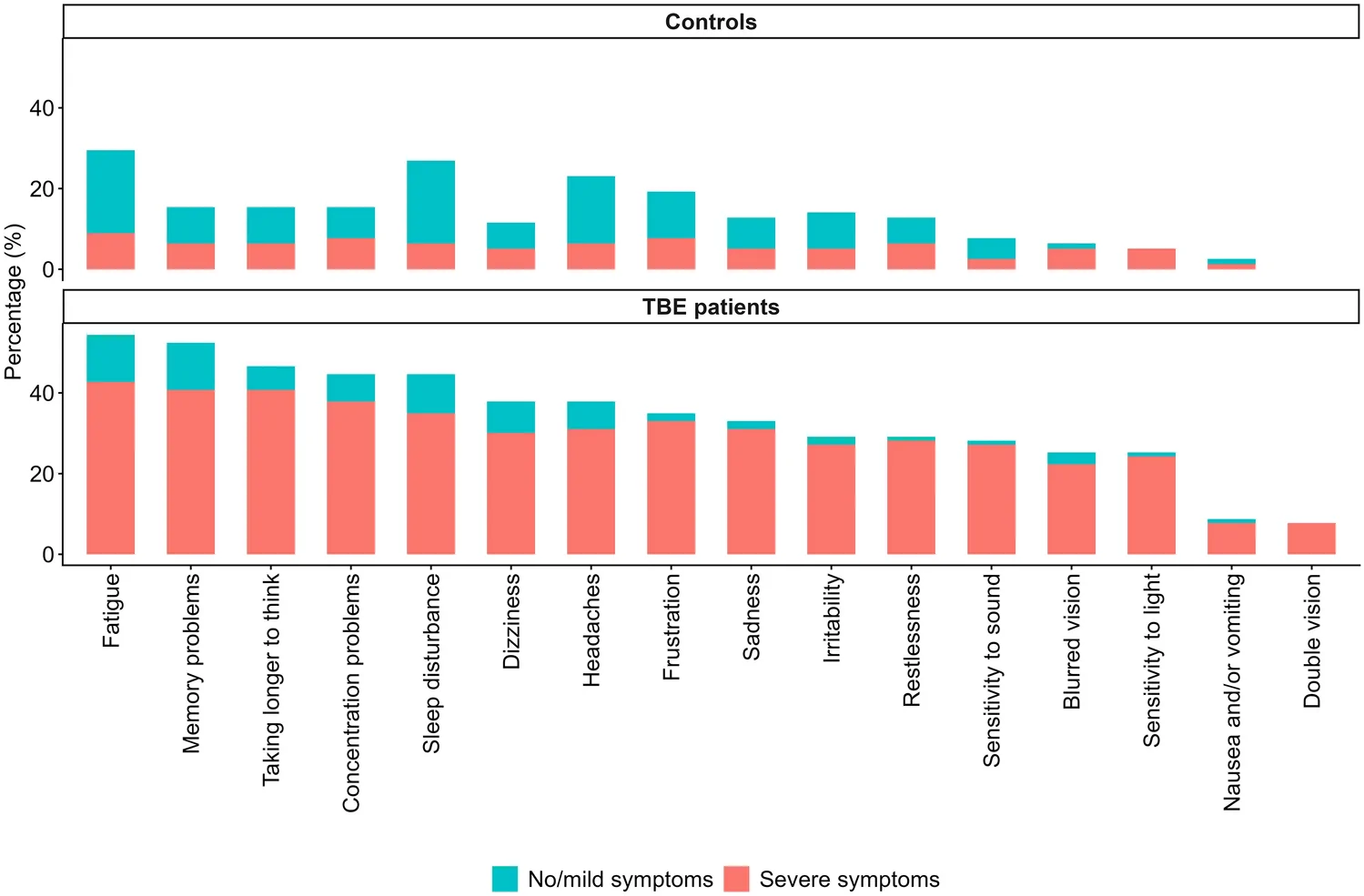
Distribution of persistent RPQ symptoms at 12 months in TBE patients (*n* = 101) versus controls (*n* = 78), *RPQ* rivermead post-concussion symptoms questionnaire. Higher scores indicate more symptoms

For everyday memory, patients scored higher than controls on the EMQ-R (mean 13.8 (SD 12.0) vs. 7.7 (SD 7.7); *p* < 0.001; Cohen`s d = − 0.60. Figure 3 illustrates these differences across age groups, showing median EMQ-R scores for patients and controls within each age group. Symptom burden on the RPQ showed the largest group difference: mean RPQ scores were 16.8 (SD 15.6) in patients versus 6.0 (SD 7.4) in controls (*p* < 0.001; Cohen`s d = − 0.84). Figure 4 shows the distribution of persisting symptoms at 12 months.

The mean BRIEF-A GEC T-scores were 53.2 (SD 12.9) in patients at 12-month and 50.9 (SD 8.9) in controls, with no significant group difference (*p* = 0.170). Across the nine BRIEF-A subscales, patients tended to have numerically higher scores on most domains (Supplementary Table 2), but these differences were non-significant, except for Working Memory, where patients reported greater difficulties than controls (mean 56.2 (SD 14.4) vs 50.3 (SD 8.6); *p* =0.002; Cohen’s d = − 0.48) (Fig. 2).

### Moderating or mediating factors and effect on main outcome

Controlling for possible mediators or moderating factors in MANCOVA (adjusting for age, sex, comorbidity, HADS-A, and HADS-D) showed that the overall significant difference between patients and controls remained unchanged (*p*: < 0.001 partial *η*^2^: 0.190. HADS-A, HADS-D, and sex were significant covariates for the combined PROMs (all *p* ≤ 0.010, Supplementary Table 3b).

Follow-up ANCOVAs, using the same covariates, showed that HADS-A, HADS-D were significant mediators of impairment for all three PROMs (all *p* ≤ 0.015). The separate ANCOVAs showed that the overall sex effect was due to sex differences in RPQ only (*p* = 0.013) (Supplementary Table 3e). Within the patient group a sex difference with higher RPQ score was present in women compared to men (mean ± SD: 20.9 ± 16.8 vs. 14.3 ± 14.5; *p* = 0.035). In contrast, among controls, there was no difference between women and men (mean ± SD: 6.8 ± 8.0 vs. 4.1 ± 5.1; *p* = 0.145). When comparing patients with controls within each sex, both women and men in the patient group had significantly higher scores than their counterparts in the control group (women: 20.9 ± 16.8 vs. 6.8 ± 8.0, *p* < 0.001; men: 14.3 ± 14.5 vs. 4.1 ± 5.1, *p* = 0.002).

### Clinical relevance of the observed differences between patients at 12-month compared to controls

Based on the predefined cut-off values (Table 1) for BRIEF-A, EMQ-R and RPQ, Table 3 shows the proportions of patients and controls with clinically significant impairment and the odds ratio for impairment when compared with controls for each measure. Statistically significant group differences in the proportion above cutoff were observed for the EMQ-R and RPQ. At 12 months, patients had higher odds of clinically significant impairment on the BRIEF-A GEC than controls, but the difference was not statistically significant (Table 3). In contrast, a significantly higher proportion of patients exceeded the cutoff on the BRIEF-A Working Memory subscale.

**Table 3:** Proportion of TBE patients at 12-month follow-up and controls with clinical impairment on BRIEF-A GEC, BRIEF-A Working Memory, EMQ-R and RPQ

Instrument-measure	Patients *n*/N^a^ (%)	Controls *n*/N^a^ (%)	OR (95% CI)	p-value
BRIEF-A GEC T-score ≥ 65	18/100 (18.0%)	7/80 (8.8%)	2.29 (0.90–− 5.79)	0.080
BRIEF-A Working Memory T-score ≥ 65	28/100 (28.0%)	5/80 (6.3%)	5.83 (2.13–−15.94)	**0.001**
EMQ-R ≥ 19	31/103 (30.1%)	5/80 (6.3%)	6.46 (2.38–−17.53)	**<0.001**
RPQ ≥ 16	46/101 (45.5%)	7/78 (9.0%)	8.19 (3.44–−19.50)	**<0.001**

*BRIEF-A* behavior rating inventory of executive function – adult version, *EMQ-R* everyday memory questionnaire – revised, *RPQ* rivermead post-concussion symptoms questionnaire, *OR* odds ratio, *CI* confidence interval

^a^All values are *n*/N (%), where *n* is the number of participants scoring above the predefined clinical cutoff and N is the total number of participants with available data in each group

Statistically significant results are shown in bold

### Factors associated with persistent cognitive symptoms 12 months after TBE

We examined factors associated with persistent cognitive symptoms at 12-month follow-up, assessed with the primary outcomes in BRIEF-A GEC, EMQ-R, and RPQ. Candidate predictors included age, sex, premorbid comorbidity, CCS scores at baseline and acute disease severity.

Multivariate regression showed that female sex was related to higher RPQ score. It also suggested a negative association between younger age and EMQ-R scores (negative coefficient), but this association was not statistically significant. However, follow-up univariate regression analyses showed that younger age was significantly negatively related both to EMQ-R and RPQ. The multivariate regression analysis showed that the presence of comorbidity was associated with about 6-point higher scores of both BRIEF-A GEC and EMQ-R scores. Higher CCS symptoms score at baseline was positively associated with BRIEF-A GEC, EMQ-R, and RPQ scores (Supplementary Table 5). Analyses comparing patients with and without clinically significant impairments showed the same pattern of results as the analyses of the total sample (data not shown).

MRI data were available for 77 patients, of whom 14 (18.2%) had MRI findings suggestive of encephalitis. CT data were available for 75 patients, among whom 1 (1.3%) had a pathological CT finding. In additional analysis, we examined the association between pathological MRI findings suggesting encephalitis and BRIEF-A GEC, EMQ-R, and RPQ at 12 months. We found no significant associations between pathological MRI findings and BRIEF-A GEC T-scores (β = 1.18, *p* = 0.763), EMQ-R (β = −4.57, *p* = 0.218) or RPQ (β = 3.84, *p* = 0.417).

### Work participation and persistent objective neurological deficits

Among 62 patients engaged in full- or part-time work at baseline (Table 2), work participation data at 3 months were available for 43. Of these, 35/43 (81.4%) had been on sick leave: 15/35 (42.9%) on full-time sick leave throughout, 15/35 (42.9%) transitioning from full-time to part-time, and 5/35 (14.3%) on part-time sick leave only. At 12 months, work participation data were available for 58 patients. Between 3 and 12 months, 27/58 (46.6%) reported sick leave. Of these, 6/27 (22.2%) remained on full-time sick leave at 12 months, 11/27 (40.7%) required a combination of full-time and part-time sick leave during the follow-up period, and 10/27 (37.0%) required part-time sick leave only. Patients on sick leave between 3 and 12 months had significantly higher scores at 12 months on the EMQ-R (*p* = 0.001) and the RPQ (*p* < 0.001) than those who were not on sick leave, whereas no statistically significant difference was observed between the groups on the BRIEF-A GEC (*p* = 0.220).

At 12 months, 36/109 (33.0%) patients had persisting objective neurological findings on examination; 24/36 (66.7%) had been classified with moderate and 7/36 (19.4%) with severe disease during the acute hospital stay for TBE. Among the 39 patients younger than 50 years at admission, 30/39 (76.9%) had moderate or severe acute disease, and 9/30 (30.0%) had persisting objective neurological deficits at 12 months. Of these 9 younger patients, 7/9 (77.8%) were classified with moderate and 2/9 (22.2%) with severe disease at admission. Of the 36 patients with persisting findings, 16/36 (44.4%) had deficits judged to clearly affect everyday functioning (≥ 1 variable scored with 2 points on the CCS). The most frequent persisting findings were balance disturbance and tremor, followed by pyramidal signs and paresis in the extremities. A more detailed description of persisting neurological deficits in this cohort has been reported previously [13].

### Sensitivity analyses

In sensitivity analyses using complete-case data (excluding imputed observations), and in analyses excluding participants with hypertension and allergy, effect estimates and conclusions were unchanged (data not shown).

## Discussion

This multicenter prospective cohort study is the first to explore cognitive difficulties during the first year after TBE in a Norwegian cohort, contributing to understanding the natural course of TBE. From 3 to 12 months, patients showed little change in self-reported executive function, everyday memory, and symptom burden. Most scored in the normal range, although a minority had persistent, clinically significant memory complaints and symptom burden at 12 months compared with comparable controls.

The minimal change from 3 to 12 months is consistent with stabilization rather than substantial late recovery, and the main trajectory of post-TBE complaints is largely determined within the first months after infection. This aligns with our previous findings [13], where symptom burden and objective neurological signs declined by 68% from the acute stage to 3 months, but by less than 9% from 3 to 12 months, and Nygren et al.[10], who reported that most recovery occurred early after symptom onset. Persistent cognitive symptoms after TBE are consistent with observations from other encephalitides, where long-term neurological, cognitive and behavioral sequelae occur in up to 50% of survivors [35]. Similar long-term cognitive impairments have been reported after autoimmune encephalitis [36], suggesting that chronic cognitive morbidity is a common feature across inflammatory CNS disorders.

Approximately one-third of patients met criteria for clinically significant memory impairment, and nearly half for clinically significant symptom burden, versus fewer than 10% of controls. Anxiety and depressive symptoms (HADS) were significant covariates, but adjustment for them did not change this pattern, and the group effect remained large (partial ${\eta }^{2}$ = 0.190). Thus, excess memory complaints and symptom burden in patients cannot be explained solely by emotional distress. Clinically, persistent cognitive complaints after TBE should be taken seriously and not dismissed as purely psychological. A subset of patients appears to have a genuine, TBE-related burden of executive, memory and symptom problems, and may benefit from systematic follow-up, including assessment of cognitive function.

Our findings of preserved global executive functioning but selective, persistent working-memory difficulties in adults parallel those of Fowler et al. [30], who reported that working memory was the most affected domain on BRIEF-A in children, and Veje et al. [17], who found lower self-reported scores in TBE cases on memory/learning and executive functions than in matched controls. This suggests that TBE is associated with a notable risk of residual, domain-specific cognitive difficulties across the lifespan.

Beyond overall group differences, certain patient characteristics were linked to persistent complaints at 12 months. Higher symptom scores measured on CCS at baseline were associated with all three primary outcome measures, whereas the CCS objective neurological findings—primarily reflecting somatic and focal neurological signs—did not predict long-term PROMs. Comorbidity was related to greater EMQ-R and BRIEF-A GEC scores, and female sex to greater RPQ compared with men. This suggests that long-term cognitive symptoms cluster in patients with a greater symptom burden and pre-existing health problems, and that the marked heterogeneity in long-term outcome after TBE is not randomly distributed within the patient cohort. Although disease severity has been associated with long-term sequelae after TBE in some studies [37, 38], Bogovič et al. [9] did not identify disease severity as an independent predictor of long-term outcome, and Griška et al. [11] found that neurocognitive impairment was not consistently associated with greater disease severity. In line with these observations, we found no association between disease severity and PROMs at 12 months, which may reflect limitations of current severity classifications that do not fully capture factors influencing long-term patient-reported cognitive outcomes.

Our work-participation data indicate that persistent symptoms meaningfully affect everyday functioning. Most working patients required sick leave in the first 3 months, and nearly half reported sick leave between 3 and 12 months; more than 20% were still on full-time sick leave at 12 months. Sick leave was associated with higher EMQ-R and RPQ scores, suggesting that persistent cognitive symptoms are closely linked to reduced work ability. Although we did not systematically collect data on reduced employment or job loss, the sustained need for sick leave implies that some patients experience lasting restrictions in professional and social participation.

At 12 months, one-third of patients still had objective neurological deficits, and nearly half of these were judged to affect everyday functioning, underscoring the long-term clinical impact of the disease. Objective findings in the acute phase were not associated with higher scores at 12 months on the instruments in the current study, suggesting that acute neurological signs alone are poor predictors of long-term cognitive difficulties, in line with a previous study showing weak correlations between standard acute-phase severity and later cognitive sequelae after TBE [11].

The notion that, in younger adults, high cognitive demands, greater stress, and heightened symptom awareness may increase the likelihood of noticing and reporting memory lapses, rather than reflecting objectively poor memory performance, has been described previously [39, 40]. In the Lithuanian cohort [11], long-term neurocognitive impairments after TBE were pronounced in younger individuals, even after mild disease. In our multivariable analysis, younger age showed a trend toward higher EMQ-R scores, indicating that younger participants tended to report more everyday memory problems than older participants, although this association did not reach statistical significance. In contrast, a recently published study [15], with a cohort partially overlapping the Lithuanian cohort [11], reported that cognitive impairment was more frequent among older participants. Together, these findings suggest that both younger and older adults may be vulnerable to substantial neurocognitive difficulties after TBE, and that self-reported problems in younger patients may not always be fully captured by objective test performance.

Comorbidities were associated with more cognitive problems on BRIEF-A GEC and EMQ-R at 12 months. Sensitivity analyses excluding participants with hypertension or allergy, the most prevalent comorbid conditions, yielded results essentially unchanged from the main analyses, indicating that our findings are not driven by these specific comorbidities, but reflect the overall comorbidity burden in the cohort. This aligns with a German prospective study [10], in which comorbidity was associated with an approximately 22% lower probability of recovery 18 months after symptom onset. We observed a clear sex difference within the patient group, with women reporting a higher symptom burden on RPQ than men, whereas no sex differences were seen among controls. Within each sex, patients reported more symptoms than controls. This pattern may indicate that TBE affects women differently than men, rather than reflecting a baseline sex difference in the general population, although sex differences in symptom perception and reporting must also be considered.

Our results are broadly compatible with the Lithuanian study [11], which used a standardized cognitive test battery to assess objective cognition after TBE. They found measurable neurocognitive deficits at follow-up despite overall clinical improvement, concluding that test performance alone was insufficient to capture the full picture of patient outcome. In our cohort, mean self-reported executive, memory, and symptom scores at 12 months were within normal ranges, yet a substantially higher proportion of patients than controls had clinically elevated scores on BRIEF-A, EMQ-R, and RPQ. These findings suggest that, even when group averages indicate a favorable prognosis, a considerable subgroup experience persistent, clinically relevant cognitive difficulties that may be missed unless both performance-based neuropsychological tests and PROMs are used.

The key strengths of this study include its prospective design with predefined assessments at 3 and 12 months, the use of a well-characterized community control group, and application of validated PROMs rather than non-standardized symptom checklists. Few studies have examined cognitive outcomes after TBE using well-characterized controls, and only a small number have applied standardized neuropsychological tests or PROMs in comparison with controls [9, 11, 17]. The control group was recruited from the patients’ social networks, rather than from a general volunteer population, which raises concern about potential selection bias. However, detailed sociodemographic data (including education, employment, and comorbidities) were collected for both groups, allowing direct comparison between them. Notably, BRIEF-A GEC mean scores in the control group were at the normative mean, rather than lower than average, as is often seen in highly selected healthy volunteer samples. This supports the representativeness of our controls. The follow-up period in the current study is relatively long for TBE, supported by a Slovenian study that found no overall improvement in postencephalitic syndrome after 12 months [9]. Loss to follow-up was limited, reducing attrition bias, and sensitivity analyses using complete-case data that yielded results similar to the main analyses. Adjustment for anxiety and depressive symptoms (HADS) reduced confounding by emotional distress and shows that the observed complaints are not explained solely by anxiety or depression.

Limitations include the reliance on self-reported questionnaires to assess cognitive outcomes. These instruments capture subjective cognitive complaints, which are known to correlate modestly with performance on formal cognitive tests [41]. Accordingly, our results should be interpreted as reflecting perceived cognitive distress and everyday executive difficulties rather than objectively verified cognitive deficits. However, such limitations are probably similar between patients and controls and are therefore unlikely to account for the observed group differences. Although we adjusted for a range of sociodemographic, premorbid, and affective variables, residual confounding from unmeasured factors (e.g. premorbid cognitive ability, personality traits) cannot be excluded. Finally, the sample size limited power for some subgroup analyses, and our findings regarding effect modifiers (e.g. age, sex, comorbidity, and acute severity) should therefore be interpreted with caution and replicated in larger cohorts. In addition, MRI was performed when clinically indicated, using non-uniform clinical protocols, which constrain inferences about structural correlates of persistent symptoms. Nevertheless, systematic, expert evaluation of the available MRI scans will be important to better characterize imaging findings and their clinical relevance.

This study suggests that TBE can lead to persistent cognitive problems one year after acute disease, even when average scores at group level are within normal range. Clinicians should not assume full recovery after “uncomplicated” TBE. In a routine clinical setting, cognitive follow-up could feasibly include a short, structured interview focusing on attention, memory and executive functioning, in combination with brief standardized tests and validated questionnaires for everyday executive problems. Such an approach may improve detection of clinically meaningful cognitive sequelae that are not evident on neurological examination alone.

Although younger age was only associated with a non-significant trend toward higher EMQ-R scores in our cohort, previous studies have suggested that younger patients may be vulnerable to long-term neurocognitive difficulties after TBE. In the Lithuanian cohort, younger adults had pronounced impairments in attention/vigilance and working memory, even after mild TBE [11]. A more recent biomarker-based study found that cognitive impairment after TBE was more frequent among older and less educated patients [15]. Taken together, these findings indicate that both younger and older adults may be vulnerable to substantial neurocognitive difficulties after TBE, and that self-reported problems in younger patients may not be fully captured by objective test performance. Clinicians should therefore maintain a high degree of awareness for both younger and older patients—particularly those with comorbidities or high acute disease burden and consider extended follow-up, cognitive rehabilitation, or workplace/educational adaptations. Although symptoms of anxiety and depression emerged as significant mediators, the present study suggests that they have limited explanatory value for who continues to experience cognitive difficulties.

By documenting long-term cognitive problems in a prospective, controlled study, our findings strengthen the evidence that TBE is often followed by persistent cognitive impairment and reinforce the rationale for TBE vaccination in endemic regions. Future studies should investigate whether patient-specific immune responses, assessed through relevant biomarkers, influence differences in TBE course and severity.

In conclusion, TBE patients showed little change in self-reported executive functioning, everyday memory, and symptom burden between 3 and 12 months, suggesting stabilization rather than substantial late recovery. At 12 months, mean scores were within or close to normal range, indicating a generally favorable group prognosis. However, a substantially larger proportion of patients than controls reported clinically elevated scores on working-memory aspects of executive function, everyday memory, and post-TBE symptoms, reflecting remaining cognitive difficulties and symptom burden. Persistent complaints clustered in patients who had comorbidities and greater acute disease burden, and these group differences remained after adjustment for anxiety and depressive symptoms. Thus, most patients recover early and remain stable, but a clinically relevant minority—identifiable by risk profile—experience enduring cognitive difficulties that warrant targeted follow-up and rehabilitation and reinforce the importance of preventive TBE vaccination in endemic areas.

## Supplementary Information

Below is the link to the electronic supplementary material.

Supplementary file1 (DOCX 62 KB)

## Data Availability

The datasets generated during and/or analyzed during the current study are not publicly available because the study is still ongoing; however, data are available from the corresponding author upon reasonable request. The data were handled in compliance with the General Data Protection Regulation (GDRP) requirements. The data are stored on TSD (Tjeneste for Sensitive Data) facilities owned by the University of Oslo, operated and developed by the TSD service group at the University of Oslo, IT-Department (USIT; tsd-drift@usit.uio.no).
