# Supplementary material for: Long-term patient-reported neurocognitive and functional outcomes after tick-borne encephalitis: a prospective controlled multicenter cohort study
Source: J Neurol. 2026 Aug 27;273(9):554. doi: 10.1007/s00415-026-14095-3 (PMC13521988; doi:10.1007/s00415-026-14095-3)
Supplement: Supplementary file 1 [file 415_2026_14095_MOESM1_ESM.docx]

**Supplementary table 1** Comorbidities and medication use in patients at admission and in controls

| **Variable** | **TBE patients  *n* = 109** | **Controls  *n* = 80** | ***p*‑value** |
| --- | --- | --- | --- |
| **Any comorbidity, yes** | 56 (51.4) | 52 (65) | 0.061 |
| Hypertension | 17 (15.6) | 14 (17.5) |  |
| Allergy | 15 (13.8) | 13 (16.3) |  |
| Chronic heart disease | 10 (9.2) | 1 (1.3) |  |
| Depression and/or anxiety | 9 (8.3) | 5 (6.3) |  |
| Diabetes | 7 (6.4) | 1 (1.3) |  |
| Chronic Obstructive Pulmonary Disease | 7 (6.4) | 2 (2.5) |  |
| Hypothyroidism | 6 (5.5) | 4 (5) |  |
| Cancer (Current/ Past) | 2 (1.8) | 8 (10) |  |
| Fibromyalgia | 3 (2.8) | 5 (6.3) |  |
| **Any medication, yes** | 57 (52.3) | 36 (45.0) | 0.322 |
| For cardiovascular diseases; including antihypertensives, anticoagulants, heart failure | 22 (20.2) | 20 (25.0) |  |
| For allergy | 10 (9.2) | 10 (12.5) |  |
| Hormonal medication: thyroid disorders | 6 (5.5) | 4 (5.0) |  |
| Hormonal medication:(e.g., oral contraceptives,  medication for menopause, other hormonal disorders) | 4 (3.7) | 9 (11.3) |  |
| For chronic ostructive pulmonary disease | 7 (6.4) | 2 (2.5) |  |
| Antidiabetic medication: Oral Tablets or Insulin | 5 (4.6) | 1 (1.3) |  |
| For pain, not specified | 5 (4.6) | 3 (3.8) |  |
| For mental illness, not specified | 2 (1.8) | 2 (2.5) |  |
| For neurological disorders, not specified | 2 (1.8) | 1 (1.3) |  |
| For cancer, active treatment | 1 (0.9) | 0 |  |

Data are presented as *n* (%)

**Supplementary Table 2** Cognitive function and symptom burden at 3 and 12-month, and patients at 12-month versus controls Mean and SD

| PROM | 3 months | 12 months | Control | p-value  3 vs 12 mo | p-value  12 mo vs control | Effect size | |
| --- | --- | --- | --- | --- | --- | --- | --- |
|  |  |  |  |  |  | Cohen’s d (95%CI)^*, †^  3 vs 12 mo | Cohen’s d (95%CI)^**, †^  12 mo vs control |
| BRIEF-A | | | | | | | |
| *n* | 76 | 100 | 80 | 71 |  |  |  |
| BRIEF-A GEC | 53.8 (12.4) | 53.2 (12.9) | 50.9 (8.9) | 0.540 | 0.170 | -0.04 (-0.17, 0.09) | -0.21 (-0.50, 0.09) |
| BRIEF-A subscales | | | | | | | |
| Inhibit | 50.5 (9.0) | 49.9 (9.0) | 48.4 (7.7) | 0.642 | 0.239 | -0.04 (-0.19, 0.11) | -0.18 (-0.47,0.12) |
| Shift | 52.2 (11.7) | 51.6 (11.9) | 51.0 (9.4) | 0.398 | 0.710 | -0.08 (-0.26, 0.10) | -0.06 (-0.35, 0.24) |
| Emotional Control | 51.9 (11.9) | 52.0 (11.5) | 49.6 (9.3) | 0.869 | 0.141 | 0.01 (-0.15, 0.18) | -0.22 (-0.52, 0.08) |
| Self-Monitor | 48.1 (10.1) | 47.6 (10.4) | 47.8 (8.7) | 0.840 | 0.883 | 0.02 (-0.14, 0.17) | 0.02 (-0.27, 0.32) |
| Initiate | 56.4 (12.6) | 55.5 (13.1) | 53.3 (9.4) | 0.215 | 0.196 | -0.10 (-0.25, 0.06) | -0.19 (-0.48, 0.10) |
| Working Memory | 55.4 (13.9) | 56.2 (14.4) | 50.3 (8.6) | 0.501 | **0.002** | 0.05 (-0.10, 0.21) | -0.48 ( -0.78, -0.18) |
| Plan/Organize | 54.8 (13.0) | 53.9 (12.6) | 52.7 (9.7) | 0.307 | 0.500 | -0.09 (-0.06, 0.08) | -0.10 (-0.40, 0.19) |
| Task Monitor | 54.2 (11.9) | 53.6 (12.2) | 53.7 (9.4) | 0.559 | 0.978 | -0.06 (-0.25, 0.13) | 0.004 (-0.29, 0.30) |
| Organization of Materials | 51.5 (10.7) | 51.7 (10.9) | 50.4 (9.4) | 0.818 | 0.413 | 0.02 (-0.11, 0.15) | -0.12 (-0.42, 0.17) |
| The Everyday Memory Questionnaire (EMQ) | | | | | | | |
| *n* | 78 | 103 | 80 | 73 |  |  |  |
| EMQ-R total score  (score 0-52) | 14.2 (12.0) | 13.8 (12.0) | 7.7 (7.7) | 0.761 | **<0.001** | -0.03 (-0.19, 0.14) | -0.60 (-0.90, -0.30) |
| Rivermead Post Concussion Symptoms Questionnaire (RPQ) | | | | | | | |
| n | 79 | 103 | 78 | 74 |  |  |  |
| RPQ total (score 0-64) | 17.2 (14.1) | 16.8 (15.6) | 6.0 (7.4) | 0.111 | **<0.001** | -0.11 (-0.24, 0.02) | -0.84 (-1.15, -0.53) |
| Hospital anxiety and depression scale (HADs) | | | | | | | |
| *n* | 80 | 106 | 80 | 78 |  |  |  |
| HAD-A | 5.1 (4.1) | 4.8 (4.0) | 4.2 (3.4) | 0.217 | 0.246 | -0.10 (-0.27, 0.06) | -0.17 (-0.47, 0.12) |
| HAD-D | 4.1 (3.7) | 4.3 (4.2) | 2.6 (3.3) | 0.882 | **0.004** | 0.01 (-0.16, 0.19) | -0.43 (-0.73, -0.14) |

*BRIEF-A* behavior rating inventory of executive function -adult version, *GEC* global executive composite, *EMQ-R* everyday memory questionnaire - revised, *RPQ* rivermead post-concussion symptoms questionnaire, *HADS-A* hospital anxiety and depression scale–anxiety subscale, *HADS-D* hospital anxiety and depression scale–depression, *CI* confidence interval
Statistically significant results are shown in bold

^*^Effect sizes (Cohen’s d) were calculated as the mean score at 12 months minus the mean score at 3 months, divided by the pooled standard deviation
^**^Effect sizes (Cohen’s d) were calculated as the mean score of control minus the mean score of patients at 12 months, divided by the pooled standard deviation
^†^ Effect size was assessed using Cohen's d. Absolute values of Cohen's d were interpreted as negligible (<0.20), small (0.20–0.49), medium (0.50–0.79), and large (≥0.80)

**Supplementary Table 3**

**Supplementary Table 3a** Repeated-measures multivariate analyses comparing the change from 3 to 12 month on square root transformed EMQ-R, RPQ, and BRIEF‑A GEC outcomes, with and without covariate adjustment.

| Source | Wilks’ Λ | F | df | *p*-value | Partial η² |
| --- | --- | --- | --- | --- | --- |
| Repeated measure MANOVA (unadjusted) | | | | | |
| Time (3 vs. 12 months) | 0.928 | 1.75 | (3,68) | 0.165 | 0.072 |
| Repeated measure MANCOVA adjusted for age, sex, disease severity, comorbidity, HADS‑A and HADS‑D | | | | | |
| Time (3 vs. 12 months) | 0.888 | 2,594 | (3, 62) | 0.060 | 0.112 |
| Age | 0.871 | 3.051 | (3, 62) | **0.035** | 0.129 |
| Sex | 0.873 | 3.016 | (3, 62) | **0.037** | 0.127 |
| Disease severity | 0.962 | 0.807 | (3, 62) | 0.495 | 0.038 |
| Comorbidity | 0.971 | 0.624 | (3, 62) | 0.602 | 0.029 |
| HADS-D | 0.940 | 1.320 | (3, 62) | 0.276 | 0.060 |
| HADS-A | 0.926 | 1,641 | (3, 62) | 0.189 | 0.074 |

*MANOVA* multivariate analysis of variance, *MANCOVA* multivariate analysis of covariance, *BRIEF-A* behavior rating inventory of executive function-adult version, *GEC* global executive composite, *EMQ-R* everyday memory questionnaire – revised, *RPQ* rivermead post-concussion symptoms questionnaire, *HADS-A* hospital anxiety and depression scale–anxiety subscale, *HADS-D* hospital anxiety and depression scale–depression subscale
Partial η² represents effect size. Outcomes were square‑root‑transformed to reduce skewness. Analyses were restricted to participants with complete data at both 3-and 12-months of follow-up across all outcome measures included in the multivariate model
Statistically significant results are shown in bold

**Supplementary Table 3b** Multivariate analyses comparing patients at 12 month and controls on square‑root‑transformed EMQ-R, RPQ, and BRIEF‑A GEC outcomes, with and without covariate adjustment

| Source | Wilks’ Λ | F | df | *p*-value | Partial η² |
| --- | --- | --- | --- | --- | --- |
| MANOVA (unadjusted) | | | | | |
| Case-Control group | 0.827 | 12.13 | (3,174) | **<0.001** | 0.173 |
| Univariate Follow‑Up ANOVAs^§^ | | | | | |
| EMQ-R |  | 10.63 | (1,176) | **0.001** | 0.057 |
| RPQ |  | 29.14 | (1,176) | **<0.001** | 0.142 |
| BRIEF A-GEC |  | 1.51 | (1,176) | 0.221 | 0.008 |
| MANCOVA adjusted for age, sex, comorbidity, HADS‑A and HADS‑D | | | | | |
| Case-Control group | 0.810 | 13.18 | (3,169) | **<0.001** | 0.190 |
| Age | 0.960 | 2.37 | (3,169) | 0.072 | 0.040 |
| Sex | 0.935 | 3.89 | (3,169) | **0.010** | 0.065 |
| Comorbidity | 0.990 | 0.58 | (3,169) | 0.627 | 0.010 |
| HADS-D | 0.890 | 6.89 | (3,169) | **<0.001** | 0.109 |
| HADS-A | 0.773 | 16.52 | (3,169) | **<0.001** | 0.227 |
| Univariate Follow‑Up ANCOVAs^‡, §^ | | | | | |
| EMQ-R |  | 6.28 | (1,171) | **0.013** | 0.035 |
| RPQ |  | 36.49 | (1, 171) | **<0.001** | 0.275 |
| BRIEF A- GEC |  | 0.32 | (1, 171) | 0.573 | 0.001 |

*MANOVA* multivariate analysis of variance, *MANCOVA* multivariate analysis of covariance, *BRIEF-A* behavior rating inventory of executive function-adult version, *GEC* global executive composite*, EMQ-R* everyday memory questionnaire – revised, *RPQ* rivermead post-concussion symptoms questionnaire, *HADS-A* hospital anxiety and depression scale–anxiety subscale, *HADS-D* hospital anxiety and depression scale–depression subscale, *ANCOVA* analysis of covariance
Partial η² represents effect size. EMQ-R, RPQ, and BRIEF‑A GEC outcome variables were analyzed after square‑root transformation to meet assumptions of normality.
^‡^Follow‑up ANCOVAs were conducted following a significant multivariate effect adjusted for age, sex, comorbidity, HADS-D and HADS-A.
**^§^**Detailed analysis of between-subjects effects can be found in Supplementary Table 3d and 3e
Statistically significant results are shown in bold

**Supplementary Table 3c** Marginal mean and corresponded standard error (SE) of EMQ-R, and RPQ with and without adjusted for covariates.

| Source | Group | Marginal mean ±SE^†^ | Adjusted marginal mean ±SE^‡^ | *p*-value^§^ |
| --- | --- | --- | --- | --- |
| EMQ-R | Control | 2.46 ± 0.18 | 2.60 ± 0.16 | **<0.001** |
|  | Case | 3.24 ± 0.16 | 3.14 ± 0.14 |  |
| RPQ | Control | 1.83 ± 0.22 | 1.89 ± 0.18 | **<0.001** |
|  | Case | 3.42 ± 0.19 | 3.37 ± 0.15 |  |

*SE* Standard Error, *EMQ-R* everyday memory questionnaire – revised, *RPQ* rivermead post-concussion symptoms questionnaire
^†^ Marginal means and SEs were obtained from ANOVA on the square‑root–transformed scale
^‡^Adjusted marginal means and SEs were obtained from ANCOVA adjusted for age, sex, comorbidity, severity of disease, HADS-A and HADS-D
**^§^** The p-values for the differences between cases and controls remained unchanged for with and without adjustment for covariates
Statistically significant results are shown in bold

**Supplementary Table 3d** Comparison of Cognitive outcomes between patients at 12 months follow-up and controls

| Cognitive outcome | Source | Type III Sum of square | df | Mean square | F | *p*-value | Partial η² |
| --- | --- | --- | --- | --- | --- | --- | --- |
| EMQ-R | Model | 26.51 | 1 | 26.51 | 10.63 | **0.001** | 0.057 |
|  | Case control | 26.51 | 1 | 26.51 | 10.63 | **0.001** | 0.057 |
|  | Error | 439.12 | 176 | 2.49 |  |  |  |
|  | Total |  | 177 | 2.63 |  |  |  |
|  | R-squared=0.057, adjusted R-squared=0.052 | | | | | | |
| RPQ | Model | 110.54 | 1 | 110.54 | 29.14 | **<0.001** | 0.142 |
|  | Case control | 110.54 | 1 | 110.54 | 29.14 | **<0.001** | 0.142 |
|  | Error | 667.54 | 176 | 3.79 |  |  |  |
|  | Total | 778.24 | 177 | 4.40 |  |  |  |
|  | R-squared=0.142, adjusted R-squared=0.137 | | | | | | |
| BRIEF-A GEC | Model | 0.87 | 1 | 0.87 | 1.51 | 0.221 | 0.008 |
|  | Case control | 0.87 | 1 | 0.87 | 1.51 | 0.221 | 0.008 |
|  | Error | 101.76 | 176 | 0.58 |  |  |  |
|  | Total | 102.63 | 177 | 0.78 |  |  |  |
|  | R-squared=0.009, adjusted R-squared=0.003 | | | | | | |

*EMQ-R* everyday memory questionnaire – revised, *RPQ* rivermead post-concussion symptoms questionnaire, *BRIEF-A* behavior rating inventory of executive function-adult version, *GEC* global executive composite
Analysis of between-subjects effects from ANOVAs corresponding to the results in table 3b. Outcomes were square‑root‑transformed
Statistically significant results are shown in bold

**Supplementary Table 3e** Comparison of Cognitive outcomes between patients at 12 months follow-up and controls adjusted for age, sex, comorbidity, HADS-A and HADS-D.

| Cognitive outcome | Source | Type III Sum of square | df | Mean square | F | *p*-value | Partial η² |
| --- | --- | --- | --- | --- | --- | --- | --- |
| EMQ-R | Model | 175.62 | 6 | 29.27 | 17.26 | **<0.001** | 0.377 |
|  | Case control | 10.65 | 1 | 10.65 | 6.28 | **0.013** | 0.035 |
|  | Age | 2.94 | 1 | 2.94 | 1.73 | 0.190 | 0.010 |
|  | Sex | 0.89 | 1 | 0.89 | 0.52 | 0.471 | 0.003 |
|  | Comorbidity | 1.08 | 1 | 1.08 | 0.64 | 0.426 | 0.004 |
|  | HADS-A | 10.20 | 1 | 10.20 | 6.01 | **0.015** | 0.033 |
|  | HADS-D | 49.01 | 1 | 49.01 | 28.90 | **<0.001** | 0.145 |
|  | Error | 290.02 | 171 | 1.70 |  |  |  |
|  | Total | 465.64 | 177 | 2.63 |  |  |  |
|  | R-squared=0.377, adjusted R-squared=0.355 | | | | | | |
| RPQ | Model | 404.65 | 6 | 67.44 | 30.87 | **<0.001** | 0.520 |
|  | Case control | 79.72 | 1 | 79.72 | 26.49 | **<0.001** | 0.176 |
|  | Age | 1.95 | 1 | 1.95 | 0.89 | 0.347 | 0.005 |
|  | Sex | 13.88 | 1 | 13.88 | 6.35 | **0.013** | 0.036 |
|  | Comorbidity | 3.70 | 1 | 3.70 | 1.69 | 0.195 | 0.010 |
|  | HADS-A | 42.83 | 1 | 42.83 | 19.61 | **<0.001** | 0.103 |
|  | HADS-D | 60.23 | 1 | 60.23 | 27.57 | **<0.001** | 0.139 |
|  | Error | 373.59 | 171 | 2.18 |  |  |  |
|  | Total | 778.24 | 177 | 4.40 |  |  |  |
|  | R-squared=0.520, adjusted R-squared=0.503 | | | | | | |
| BRIEF-A GEC | Model | 37.21 | 6 | 6.20 | 26.21 | **<0.001** | 0.362 |
|  | Case control | 0.12 | 1 | 0.12 | 0.32 | 0.573 | 0.002 |
|  | Age | 0.51 | 1 | 0.51 | 1.33 | 0.251 | 0.008 |
|  | Sex | 0.42 | 1 | 0.42 | 1.11 | 0.295 | 0.006 |
|  | Comorbidity | 0.10 | 1 | 0.10 | 0.25 | 0.617 | 0.001 |
|  | HADS-A | 2.71 | 1 | 2.71 | 7.07 | **0.009** | 0.040 |
|  | HADS-D | 14.09 | 1 | 14.09 | 36.83 | **<0.001** | 0.177 |
|  | Error | 65.43 | 174 | 0.38 |  |  |  |
|  | Total | 102.63 | 177 | 0.58 |  |  |  |
|  | R-squared=0.363, adjusted R-squared=0.340 | | | | | | |

*EMQ-R* everyday memory questionnaire – revised, *RPQ* rivermead post-concussion symptoms questionnaire, *BRIEF-A* behavior rating inventory of executive function-adult version, *GEC* global executive composite, *HADS-A* hospital anxiety and depression scale–anxiety subscale, *HADS-D* hospital anxiety and depression scale–depression subscale
Analysis of between-subjects effects from ANCOVAs corresponding to the results in table 3b. Outcomes were square‑root‑transformed
Statistically significant results are shown in bold

**Supplementary Table 4** Univariable linear regression analyses of predictors of PROM scores at 12 months

| Predictor | BRIEF-A GEC *n*=100  β (95 % CI) | *p-*value | EMQ-R  *n*=103  β (95 % CI) | *p*-value | RPQ *n*=103  β (95 % CI) | *p*-value |
| --- | --- | --- | --- | --- | --- | --- |
| Age | -0.10 (-0.27, 0.06) | 0.216 | -0.17 (-0.32, 0.02) | **0.025** | -0.20 (-0.40, -0.01) | **0.044** |
| Sex (female vs male) | 2.00 (-3.27, 7.26) | 0.454 | -0.25 (-5.11, 4.62) | 0.920 | 6.67 (0.48, 12.87) | **0.035** |
| Any comorbidity (yes vs no) | 5.07 (0.04, 10.10) | **0.048** | 3.73 (-0.93, 8.39) | 0.115 | 3.76 (-2.34, 9.85) | 0.224 |
| Disease severity |  |  |  |  |  |  |
| Moderate vs mild | 3.06 (-3.35, 9.48) | 0.345 | 3.47 (-2.29, 9.22) | 0.235 | 1.77 (-5.78, 9.33) | 0.643 |
| Severe vs mild | 4.74 (-4.30, 13.77) | 0.301 | -0.15 (-8.41, 8.11) | 0.972 | -0.19 (-11.04, 10.66) | 0.972 |
| CCS symptom score | 0.77 (0.26-1.28) | **0.003** | 0.81 (0.35-1.27) | **0.001** | 1.37 (0.80-1.95) | **<0.001** |
| CCS objective findings | 0.26 (-0.28-0.80) | 0.341 | 0.19 (-0.31- 0.68) | 0.449 | 0.23 (-0.41-0.88) | 0.476 |

*BRIEF-A* behavior rating inventory of executive function-adult version, *GEC* global executive composite*, EMQ-R* everyday memory questionnaire – revised, *RPQ* rivermead post-concussion symptoms questionnaire, *CCS* composite clinical score, *CI* confidence interval
Reference categories: male sex, mild disease, no comorbidity. Higher scores indicate more severe symptoms or executive/memory problems
Statistically significant results are shown in bold

**Supplementary Table 5. Multivariable linear regression analyses of predictors of PROM scores at 12 months**

| Predictor | BRIEF‑A GEC  *n* = 99  β (95 % CI) | *p*-value | EMQ total  *n* = 102  β (95 % CI) | *p*-value | RPQ total  *n* = 102  β (95 % CI) | *p*-value |
| --- | --- | --- | --- | --- | --- | --- |
| Age | -0.10 (-0.30, 0.09) | 0.296 | -0.17 (-0.34, 0.01) | 0.061 | -0.14 (-0.36, 0.08) | 0.209 |
| Sex, female | 1.64 (-3.59, 6.86) | 0.536 | -0.97 (-5.64, 3.70) | 0.680 | 6.51 (0.65, 12.37) | **0.030** |
| Any comorbidity | 5.67 (0.25, 11.09) |  | 6.35 (1.51, 11.18) | **0.011** | 4.73 (-1.33, 10.80) | 0.125 |
| Disease severity |  |  |  |  |  |  |
| Moderate vs mild | 2.30 (-4.69, 9.30) | 0.515 | 2.33 (-3.86, 8.52) | 0.456 | -0.92 (-8.69, 6.84) | 0.814 |
| Severe vs mild | 1.59 (-9.52, 12.70) | 0.776 | -4.61 (-14.45, 5.24) | 0.355 | -4.57 (-16.93,7.79) | 0.465 |
| CCS symptoms score | 0.62 (0.04-1.20) | **0.035** | 0.56 (0.04, 1.08) | **0.034** | 1.19 (0.54, 1.84) | **< 0.001** |
| CCS objective findings | 0.08 (-0.61-0.77) | 0.818 | 0.23 (-0.39, 0.85) | 0.458 | 0.15 (-0.63, 0.92) | 0.706 |

*BRIEF-A* behavior rating inventory of executive function-adult version, *GEC* global executive composite*, EMQ-R* everyday memory questionnaire – revised, *RPQ* rivermead post-concussion symptoms questionnaire, *CCS* composite clinical score, *CI* confidence interval
Values are regression coefficients $\beta$ with 95 % confidence intervals (CI) and p-values.
Reference categories: male sex, mild disease, no comorbidity. Higher scores indicate more severe symptoms or executive/memory problems
Statistically significant results are shown in bold

**Handling of missing questionnaire data and internal consistency**

**The Behavior Rating Inventory of Executive Function – Adult version (BRIEF‑A)**

Missing responses were handled according to the BRIEF‑A manual, where one missing item within a subscale was given the lowest of three possible values (one). This rule was applied to two scores at the 3‑month follow‑up, six scores at 12‑month follow‑up and for eight controls. If more than one item was missing within a subscale, the subscale was excluded from the analysis (3‑month follow‑up, *n* = 2; 12‑month follow‑up, *n* = 3; controls, *n* = 2).

Cronbach’s α was 0.96 (3-month), 0.98 (12-month), and 0.96 in controls.

**Everyday memory was assessed using the Everyday Memory Questionnaire–Revised (EMQ‑R)**

Missing questionnaire items in EMQ-R were replaced according to the following rules; when a form had only one missing item, we imputed the mean of the completed items for that respondent (3‑month follow‑up, *n* = 2; controls, *n* = 2). There were no missing items beyond a single item. One participant attended the follow‑up visit but did not respond to any of the EMQ-R questions; therefore, this questionnaire was excluded from the analysis.

Cronbach’s α was 0.94 (3‑month) follow‑up, 0.95 (12‑month), and 0.91 in controls.

**The Rivermead Post‑Concussion Symptoms Questionnaire (RPQ)**

Missing questionnaire items were replaced according to the following rules; if the participant had provided the same response during the clinical investigation and it was recorded in CCS, that result was used if it was comparable to the Likert scale (12-months follow-up, *n* = 4). If one item was missing and no clinical value was available, we imputed the mean of the completed items (12-month follow‑up, *n* = 3; controls, *n* = 1). There were no missing items beyond a single item. Two participants from control group did not respond to any of the RPQ questions; therefore, they were excluded from the analysis.

Cronbach’s α was 0.94 (3 months), 0.95 (12 months), and 0.86 in controls for the RPQ.
